# Trends in Exposure to Diesel Particulate Matter and Prevalence of Respiratory Symptoms in Western Australian Miners

**DOI:** 10.3390/ijerph17228435

**Published:** 2020-11-14

**Authors:** Krassi Rumchev, Dong Van Hoang, Andy Lee

**Affiliations:** 1 School of Public Health, Curtin University, Perth 6120, Australia; Andy.Lee@curtin.edu.au; 2 Department of Epidemiology and Prevention, Center for Clinical Sciences, National Center for Global Health and Medicine, Tokyo 162-8655, Japan; vhoang@hosp.ncgm.go.jp

**Keywords:** diesel particulate matter, underground mines, respiratory symptoms, Australia

## Abstract

Diesel-powered equipment is used frequently in the mining industry. They are energetically more efficient and emit lower quantities of carbon monoxide and carbon dioxide than the gasoline equipment. However, diesel engines release more diesel particulate matter (DPM) during the combustion process which has been linked to harmful health effects. This study assessed the trends in DPM exposure and the prevalence of respiratory symptoms among Western Australian miners, using the available secondary data collected from 2006 to 2012. The data consisted of elemental carbon (EC) concentrations and information on miner’s respiratory symptoms. The measured EC concentrations from *n* = 2598 miners ranged between 0.01 mg/m^3^ and 1.00 mg/m^3^ and tended to significantly decrease over the study period (*p* < 0.001). Underground mine workers were exposed to significantly higher (*p* < 0.01) median EC concentrations of 0.069 mg/m^3^ (IQR 0.076) when compared to surface workers’ 0.038 mg/m^3^ (IQR 0.04). Overall, 29% of the miners reported at least one respiratory symptom, with the highest frequency recorded for cough (16%). Although the exposure levels of DPM in the mining industry of Western Australia have declined over the study period, they are still high and adhering to stringent occupational standard for DPM is recommended.

## 1. Introduction

Diesel-powered vehicles provide the main source for transport in the mining industry. They are more efficient, energetically more effective, and emit less carbon monoxide and carbon dioxide than their gasoline counterpart [1]. Despite all the advantages, diesel-powered engines emit more diesel particulate matter (DPM) than gasoline equipment during the combustion process [2,3,4]. DPM is composed of elemental carbon (EC) onto which organic carbon (OC) compounds and other particles (unburnt fuel, lubricant droplets, metallic additives, etc.) are adsorbed. The sum of OC and EC in the DPM fraction represents the total carbon (TC). TC is frequently used as an indicator of DPM exposure, but OC from other sources, such as cigarette smoke, oil mist, and other fuels, may interfere with the analysis [5,6]. Therefore, measurements of EC have been proposed to reduce the influence of OC contamination in DPM assessments [5,7,8].

Approximately 80–95% of the diesel particle mass is in the fine particle size range (≤2.5 μm), with a mean particle aerodynamic diameter of about 0.2 μm [8]. Particles with aerodynamic diameters >2.5 μm (i.e., >PM_2.5_) tend to be retained in the upper portions of the respiratory tract, whereas particles with diameters <2.5 μm (i.e., PM_2.5_) are deposited in all areas, especially into the lower part of the respiratory tract, including the alveolar region [8,9,10,11]. These fine and ultrafine particles have a very large surface area per gram mass, which enables them to adsorb and ultimately transport inorganic and organic compounds into the lung [8,9,12,13].

Short-term health effects from exposure to DPM include throat and bronchial irritation, cough, phlegm, and neurophysiological symptoms [8,14]. Combined experimental and human chronic exposure studies show evidence of long-term adverse DPM impact on the respiratory and cardiovascular systems [8,15,16,17,18,19]. Based on the proven genotoxicity of its constituents, diesel exhaust has been confirmed as mutagenic and carcinogenic to humans by the World Health Organization in 2010 [20]. In June 2012, the International Agency for Research on Cancer (IARC) classified diesel engine exhaust as a Group 1 carcinogen to humans.

Mine workers are exposed to far higher DPM concentrations than other professionals. Coble and colleagues [21] found the average EC level measured for underground miners ranged from 64 to 195 µg/m^3^, which is approximately twice the level measured for surface workers (38 to 71 µg/m^3^). The EC exposure levels for truck, bus, and taxi drivers were generally between 1–10 µg/m^3^, but for fire fighters they were mostly non-detectable [22]. In Australia, the EC levels among miners ranged between 10 and 420 µg/m^3^ for coal mines and between 11 and 117 µg/m^3^ for underground metal mines [23,24] (AIOH, 2013; Irving G, 2006).

The occupational exposure limit (OEL) for diesel emission varies between and within countries. In the province of Quebec, Canada, the limit of 400 µg/m^3^ (0.40 mg/m^3^) for TC was established in 2016 [25]. The U.S. Mine Safety and Health Administration has recommended an 8-h OEL of 160 µg/m^3^ (0.16 mg/m^3^) for TC [7]. However, there is no current occupational exposure standard for DPM in Australia. The Australian Institute of Occupational Hygiene [23] has set a guideline value whereby workers exposed to DPM should be controlled to below 100 µg/m^3^ (0.1 mg/m^3^) as an 8 h time weighted average value, measured as submicron EC, which is approximately equal to 0.16 mg/m^3^ TC or 0.2 mg/m^3^ diesel particulate.

The present study investigated the trends in DPM concentrations and prevalence of respiratory symptoms among workers in Western Australian mines for the period 2006–2012. The findings can contribute for setting a standard concerning DPM exposure level in the Australian mining industry.

## 2. Materials and Methods

### 2.1. DPM Exposure Measurement

All mining companies in Western Australia were required by the government to conduct regular exposure assessments of selected contaminants among mine workers. The mining sector produces more than 50 different minerals from about 1000 operating mines in Western Australia. Most diesel engines in underground mines are used to power support equipment and not production equipment, mainly for loading and hauling operations. They can be classified under three categories: permissible diesel equipment, heavy-duty non-permissible, and light-duty non-permissible diesel equipment. Additionally, there are four types of diesel engines, with Tier 3 and 4 engines having the lowest diesel emissions, although Tier 1 and 2 engines are still used in Australia [26], as they comply with the government standard, *AS/NZS 3584 Set: 2012 Diesel Engine Systems Standards for Underground Coal Mines*.

The sampling quota was determined for each mining site to ensure a representative sample. Personal exposure monitoring was conducted four times a year at each mine site. All data were submitted electronically within six weeks and systematically recorded in the CONTAM database, which is operated and managed by the Department of Mines and Petroleum (DMP), Western Australia (WA).

EC, as a marker for DPM exposure, was measured for the mining employees from 2006 until the end of 2012 following the NIOSH Method 5040 [27]. DPM Plastic Cyclone 37 mm has been used to meet the NIOSH 5040 specifications. The SKC DPM Cassette has a built-in impactor, which performs the differentiation of DPM, where respirable dust with particle size ≥1.0 μm is screened out, leaving only those with particle size <1.0 μm collected on the filter. The sampler was attached to a personal sampling pump and clipped onto clothing in the breathing zone of mine workers. The sampling flow rate was maintained at between 2 to 4 L/min. The work shift length of monitored miners was either 8 h or 12 h.

### 2.2. Respiratory Health Surveillance

In addition to the contaminant exposure assessment, the Mines Safety and Inspection Act 1994 and Regulations 1995 required a regular respiratory health surveillance to identify those with elevated dust exposures and risk for the development of respiratory symptoms. The health surveillance questionnaire was administered after a worker had entered the Western Australian mining industry, and then periodically or as directed. The respiratory health data were stored in the MineHealth database.

Questionnaires and consent forms were administered to randomly selected mine workers by the mining companies following guidelines from the DMP. The standardized questionnaire consisted of questions including gender, age, job title, shift length and pattern, use of respirator mask during work, and current smoking status (smoker, non-smoker). Mine workers were asked to report current respiratory symptoms and occurring in the last 3 months, namely, ‘cough’ (usually cough first in the morning, during the day or at night), ‘phlegm’ (usually bring up phlegm first in the morning, during the day or at night), ‘wheeze’ (ever experience of the chest sounding wheezy or whistling), ‘breathlessness’ (either short of breath at rest or on activity), and ‘any respiratory symptom’ (having at least one of the above symptoms). Responses to the respiratory questions were binary (yes/no). Mine companies were responsible for the data collection process and entry of the hygiene and health data directly into the Safety Regulations System (SRS). The CONTAM and MineHealth database were then linked and records were matched by the DMP, WA, using each worker’s unique identifier number.

### 2.3. Occupational Grouping

There were 87 place descriptions and 129 jobs in the original dataset. To facilitate analysis, the occupation groups were classified as either ‘surface production and services’ or ‘underground mining.’ The first group consisted of occupations in which workers were not exposed or had limited exposure to heavy diesel equipment. They included a wide range of professional activities related to geologists, mobile plant operators, engineers, electricians, and mechanics. The second group comprised mine workers who were involved in mining production or development, drilling, blasting, loading, and transport operations.

### 2.4. Statistical Analysis

Characteristics of the analytic sample were described by means (SD) for continuous variables, and percentages for categorical variables. The annual median (IQR) EC concentrations were calculated and stratified by occupation (surface production and services; underground mining), mask wearing status (yes; no), and work shift length (8 h; 12 h). Prevalence of each and any respiratory symptom was calculated annually. The overall prevalence (across all years) of respiratory symptoms was then compared with respect to occupation, mask wearing, work shift length, and current smoking status (smoker; non-smoker). Logistic regression was then conducted to estimate the risk of respiratory symptom for any apparent association from the univariate analysis, with “respiratory symptom status” (0 = no; 1 = yes) as the dependent variable. The linear time trend in EC levels was tested by assigning ordinal numbers (1–7) to each year (2006–2012), then fitting a linear regression model of the EC concentration for the entire dataset (*n* = 2598). The linear time trend in the prevalence of any respiratory symptom was assessed in a similar manner. All statistical analyses were performed in RStudio for Windows version 3.2.4 (RStudio Team 2012, Boston, MA, USA).

## 3. Results

### 3.1. Study Population

The CONTAM database contained 6785 DPM records for the period 2006–2012, with a total of 124 mines being assessed for exposure to EC. After excluding (i) workers who had been surveyed in previous year(s), (ii) observations with work shift length <8 or >12 h, and (iii) those workers whose occupation was not related to mining activity, the final analytic sample comprised *n* = 2598 workers from the total *N* = 2964 eligible miners participating in the surveys (Table 1). Among them, 2471 (95%) were male workers. The mean age of the study population was 35 ± 10 years, and 93.5% were occupied in underground mining activities. The great majority of mine workers did not wear protective equipment from dust exposure (90.1%) and worked long, 12-h shifts (90.6%). Just over one-third of them were current smokers (36.7%).

### 3.2. EC Exposure

**Figure 1:**
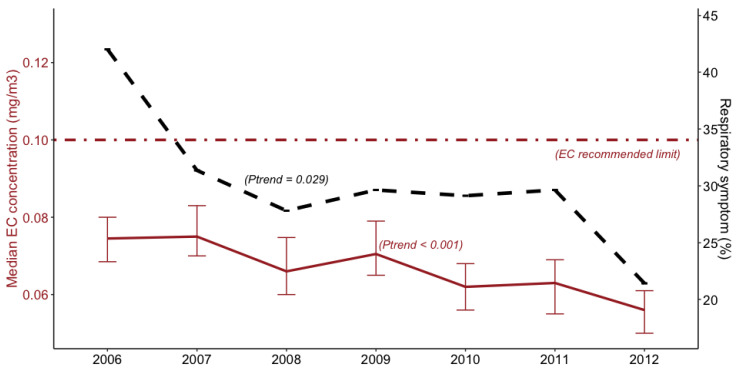
Median elemental carbon concentration (solid line) and prevalence of any respiratory symptom (dotted line) of Western Australian miners, 2006–2012.

Table 2 presents the average EC concentration by occupation, respiratory mask wearing status, and shift length. The EC exposure levels ranged between 0.01 mg/m^3^ and 1.00 mg/m^3^ and steadily declined over the study period (*p* for decreasing time trend <0.001); and the median concentrations were below the Australian guideline of 0.1 mg/m^3^ (Figure 1). The underground miners were exposed to significantly higher (median 0.069 mg/m^3^) levels of EC than those involved in surface operations (median 0.038 mg/m^3^). Stratified by respiratory protective equipment, mine workers who did not wear a respiratory mask during work were exposed to significantly higher EC concentration (median 0.07 mg/m^3^), when compared with those who used such protection equipment (median 0.04 mg/m^3^). However, it appears that the length of work shift did not have an effect on the EC exposure levels of miners.

### 3.3. Respiratory Health

Table 3 summarizes the prevalence of respiratory symptoms for mine workers over the study period. Overall, 28.6% of the miners reported at least one respiratory symptom, with the highest frequency recorded for cough (15.7%).

As shown in Figure 1, the declining time trend in the prevalence of any respiratory symptom is evident (*p* = 0.029) and in line with the reduced EC concentrations over the study period.

When stratified by occupation, shift length, respiratory mask wearing status, and smoking status (Table 4), higher frequencies of respiratory symptoms were observed for workers involved in underground mining and among those who had no protection from dust exposure, particularly for cough (*p* = 0.017 and *p* = 0.036, respectively). The prevalence of respiratory symptoms was similar between miners who worked 12 h and 8 h shifts. As expected, smokers reported significantly higher prevalence of all respiratory symptoms than their non-smoking counterparts (*p* < 0.001).

According to logistic regression analysis, the risk of cough was significantly higher (*p* = 0.025) among underground miners than surface workers after adjustment for their age and smoking status, the estimated odds ratio being 1.64 (95% confidence interval 1.08–2.58).

Table 5 shows that mine workers who reported cough and phlegm were exposed to elevated EC concentrations (*p* < 0.001), when compared to those without such symptoms. In addition, the respiratory symptoms appeared to be more common among older mine workers (Table 5).

## 4. Discussion

This study assessed exposure to EC levels among a large population of mine workers in Western Australia and their respiratory health for the period 2006–2012. A key finding was that underground miners were exposed to significantly higher EC concentrations than surface production workers over the study period, except in 2006, probably due to limited DPM monitoring of underground miners before the introduction of the exposure guideline by the Australian Institute of Occupational Hygiene in 2007 [28].

The findings indicated that 90% of workers did not wear protective equipment when working in dusty environments, which contributed to their elevated EC levels. Although the EC concentrations ranged from 0.01 to 1.00 mg/m^3^ in this study, the estimated median exposures tended to decline over the study period and were below the Australian guideline value of 0.1 mg/m^3^.

A study of metalliferous underground mining operations in Queensland [24] showed similar EC levels of 0.01 to 0.117 mg/m^3^ for underground operators. A comparison with international studies indicated that levels in WA mines were of similar or less order of magnitude as found in the mining industry elsewhere in the world. In the USA, the average EC levels measured for underground miners ranged from 0.064 to 0.19 mg/m^3^, while lower EC levels for the surface workers were measured, with a range between 0.04 to 0.07 mg/m^3^ [21]. Similar recordings were reported in a study from Canada with the mean exposure ranging from 0.04 to 0.160 mg/m^3^ [29]. In a more recent study conducted among underground miners in Sweden, EC exposure levels varied from 0.05 to 0.061 mg/m^3^ [30]. Exposure levels of EC among underground miners were measured in a US goldmine (1990s), where the diesel equipment had no emission control devices, and they ranged from 0.305 to 1.165 mg/m^3^ [31].

These wide range in EC exposure levels may be due to high variability between facilities and suggests that other determinants, such as type of diesel equipment, maintenance, and ventilation efficiencies in different mine sites, may also play a role in the exposure levels to diesel particulates. Sampling procedures could also differ between mine sites.

The median EC concentrations observed in our study are at the lower end of the exposure levels reported in most international studies. The rapid decline in EC exposure level in 2007 can be explained by the introduction of DPM guideline value in the same year [28]. Since then, mining companies have embarked on utilizing control technologies to reduce diesel emissions in underground mines. The implemented efforts for controlling diesel exhaust in the Australian mines included vehicle standards and statutory implications [32], vehicle management strategies [33], alternative diesel fuels [34], ultrasonic transducers with electrostatic perspiration filter [35], and cooled exhaust and scrubber [36].

Mining activities, both surface and underground, have been associated with adverse health conditions, including acute and chronic respiratory diseases. In this study, 29% of the interviewed miners reported at least one respiratory symptom over the six-year study period, with the highest frequency recorded for cough (15.7%), followed by phlegm (11.7%), wheeze (10.5%), and breathlessness (9.6%). Somewhat higher rates of prevalence for cough (28.3%) and breathlessness (22.3%) were found among miners in one study [37], but much lower prevalence rates had been reported in another study [38], namely, 9% and 3%, respectively. The observed decreasing trend in respiratory symptoms could be associated with the apparent reduction in EC concentration over the years, but might also be attributed to the improved illnesses prevention and management in the Australian healthcare system [39]. Meanwhile, there has been a continuing decline in the daily smoking rate among the general population, from 22.4% in 2001 to 18.9% in 2007 and 16.3% in 2011 [40]. Smoking is well recognized as an independent predictor of declines in respiratory health and significantly higher prevalence of cough and phlegm was evident among current smokers in this study. Similar findings have been established in other studies, although at lower rates [41,42]. Overall, 42% of miners who smoked reported a respiratory symptom, as compared to 21% of those who did not smoke.

Underground miners appeared to be more susceptible to respiratory illness, in line with their elevated EC exposure levels, while those who reported cough and phlegm were exposed to significantly higher EC concentrations than others without such symptoms. The present study found that the prevalence of cough among underground miners was 64% higher than that of surface workers, consistent with the findings of a previous study [43]. Moreover, our reported overall prevalence of cough (15.7%) for the miners was higher than that of the general population (12.5%) [44].

The EC concentrations found in this study are within the recommended guideline value of 0.1 mg/m^3^. However, a study by Vermeulen and colleagues [45] showed an increased risk of lung cancer at these levels. They estimated excess lung cancer deaths per 10,000 individuals for average lifetime occupational exposure levels between 0.001 and 0.025 mg/m^3^ (1–25 μg/m^3^); similar values have been recorded in our study.

The use of administrative databases was a strength of the present study, which provided access to a large data set. Similar studies have been conducted in China [46,47], in another large international study that involved a number of European countries [48], and in a joint study of Europe and Canada [49]. The outcomes of these studies clearly demonstrate the feasibility of this approach in occupational exposure assessment, although the authors of the current study acknowledge the use of existing data without involvement in the data collection process as a study limitation. The reliance on self-reported respiratory symptoms without medical diagnoses posed another limitation for this study. Nevertheless, self-report data from the respiratory health survey were used in conjunction with the quantitative DPM measurements and other information through record matching and data linkage to reduce bias and inaccuracy [50].

## 5. Conclusions

The concentrations of DPM in the mining industry of WA was still high, despite the reductions in exposure levels in recent years and the apparent declining trend in the prevalence of respiratory symptoms. The findings suggest that stringent occupational standard for DPM should be maintained in order to protect the respiratory health of mine workers.

## Figures and Tables

**Table 1:** Characteristics of the Western Australian miners, 2006–2012.

	2006 *N* = 239 *n* = 239	2007 *N* = 388 *n* = 327	2008 *N* = 430 *n* = 351	2009 *N* = 507 *n* = 435	2010 *N* = 452 *n* = 390	2011 *N* = 381 *n* = 343	2012 *N* = 567 *n* = 513	Total *N* = 2964 *n* = 2598
Age, mean (SD)	35.3 (9.3)	35.3 (9.8)	34.8 (9.7)	35.7 (9.6)	34.7 (10.1)	35.1 (10.7)	33.9 (10.1)	34.9 (9.9)
Sex, *n* (%)								
Female	6 (2.5)	15 (4.6)	21 (5.9)	12 (2.8)	21 (5.4)	23 (6.7)	29 (5.6)	127 (4.9)
Male	233 (97.5)	312 (95.4)	330 (94.0)	423 (97.2)	369 (94.6)	320 (93.3)	484 (94.3)	2471 (95.1)
Occupation group, *n* (%)								
Surface production	7 (2.9)	10 (3.1)	23 (6.6)	21 (4.8)	12 (3.1)	34 (9.9)	61 (11.9)	168 (6.5)
Underground mining	232 (97.1)	317 (96.9)	328 (93.4)	414 (95.2)	378 (96.9)	309 (90.1)	452 (88.1)	2430 (93.5)
Mask wearing, *n* (%)								
No	219 (91.6)	299 (91.4)	308 (87.7)	395 (90.8)	356 (91.3)	314 (91.5)	451 (87.9)	2342 (90.1)
Yes	20 (8.34)	28 (8.6)	43 (12.3)	40 (9.2)	34 (8.7)	29 (8.5)	62 (12.1)	256 (9.9)
Shift length, *n* (%)								
12 h	214 (89.5)	281 (85.9)	311 (88.6)	389 (89.4)	370 (94.9)	313 (91.3)	477 (93.0)	2355 (90.6)
8 h	25 (10.5)	46 (14.1)	40 (11.4)	46 (10.6)	20 (5.2)	30 (8.8)	36 (7.0)	243 (9.4)

**Table 2:** Elemental carbon exposure of Western Australian miners by occupation group, mask wearing status, and work shift length, 2006–2012.

Year	*n*	Overall	Elemental Carbon Concentration (mg/m^3^)								
			Occupation Group			Mask Wearing			Shift Length		
			Surface Production	Underground Mining	*p*	Yes	No	*p*	8 h	12 h	*p*
**2006**	239		7	232		20	219		25	214	
Median (IQR)		0.074 (0.067)	0.09 (0.455)	0.074 (0.066)	0.109	0.05 (0.037)	0.076 (0.068)	0.009	0.072 (0.07)	0.075 (0.067)	0.884
**2007**	327		10	317		28	299		46	281	
Median (IQR)		0.075 (0.085)	0.053 (0.04)	0.077 (0.085)	0.105	0.04 (0.05)	0.079 (0.086)	<0.001	0.068 (0.093)	0.077 (0.085)	0.001
**2008**	351		23	328		43	308		40	311	
Median (IQR)		0.066 (0.072)	0.045 (0.024)	0.07 (0.071)	0.001	0.05 (0.035)	0.071 (0.074)	0.007	0.058 (0.106)	0.067 (0.065)	0.665
**2009**	435		21	414		40	395		46	389	
Median (IQR)		0.07 (0.087)	0.034 (0.019)	0.073 (0.086)	<0.001	0.039 (0.055)	0.073 (0.087)	0.004	0.06 (0.062)	0.072 (0.086)	0.004
**2010**	390		12	378		34	356		20	370	
Median (IQR)		0.062 (0.065)	0.041 (0.048)	0.062 (0.065)	0.113	0.027 (0.04)	0.066 (0.061)	<0.001	0.064 (0.064)	0.062 (0.065)	0.706
**2011**	343		34	309		29	314		30	313	
Median (IQR)		0.063 (0.076)	0.035 (0.046)	0.065 (0.085)	<0.001	0.041 (0.04)	0.065 (0.084)	0.001	0.057 (0.077)	0.064 (0.075)	0.447
**2012**	513		61	452		62	451		36	477	
Median (IQR)		0.056 (0.077)	0.037 (0.038)	0.06 (0.078)	<0.001	0.04 (0.061)	0.058 (0.079)	0.07	0.105 (0.096)	0.055 (0.068)	0.087
**Total**	2598		168	2430		256	2342		243	2355	
Median (IQR)		0.066 (0.073)	0.038 (0.04)	0.069 (0.076)	<0.001	0.04 (0.044)	0.07 (0.077)	<0.001	0.066 (0.088)	0.067 (0.073)	0.521

**Table 3:** Prevalence of respiratory symptoms of Western Australian miners, 2006–2012.

Year	*N*	Respiratory Symptom, *n* (%)				
		Cough	Phlegm	Wheeze	Breathlessness	Any
2006	239	62 (25.9)	33 (13.8)	33 (13.8)	41 (17.2)	99 (41.4)
2007	327	70 (21.4)	38 (11.6)	38 (11.6)	31 (9.5)	101 (30.9)
2008	351	41 (11.7)	39 (11.1)	35 (10.0)	33 (9.4)	96 (27.4)
2009	435	66 (15.2)	57 (13.1)	45 (10.3)	48 (11.0)	127 (29.2)
2010	390	58 (14.9)	52 (13.3)	36 (9.2)	31 (7.9)	112 (28.7)
2011	343	51 (14.9)	39 (11.4)	44 (12.8)	29 (8.5)	100 (29.2)
2012	513	59 (11.5)	45 (8.8)	43 (8.4)	36 (7.0)	108 (21.1)
**Total**	2598	407 (15.7)	303 (11.7)	274 (10.5)	249 (9.6)	743 (28.6)
***p*-*trend***		0.041	0.159	0.174	0.041	0.029

**Table 4:** Prevalence of respiratory symptoms by occupation group, mask wearing status, and work shift length (*n* = 2598).

	*n*	Respiratory Symptom, *n* (%)				
		Cough	Phlegm	Wheeze	Breathlessness	Any
**Occupation group**	
Surface production	253	26 (10.3)	20 (7.9)	26 (10.3)	28 (11.1)	61 (24.1)
Underground mining	2345	381 (16.2)	283 (12.1)	248 (10.6)	221 (9.4)	682 (29.1)
*p*		0.017	0.063	0.969	0.465	0.112
**Mask wearing**						
No	2342	379 (16.2)	279 (11.9)	252 (10.8)	227 (9.7)	681 (29.1)
Yes	256	28 (10.9)	24 (9.4)	22 (8.6)	22 (8.6)	62 (24.2)
*p*		0.036	0.272	0.335	0.649	0.119
**Shift length**						
12 h	2355	361 (15.3)	274 (11.6)	252 (10.7)	225 (9.6)	676 (28.7)
8 h	243	46 (18.9)	29 (11.9)	22 (9.1)	24 (9.9)	67 (27.6)
*p*		0.168	0.973	0.493	0.962	0.766
**Smoking status**						
Smoker	953	264 (27.7)	183 (19.2)	153 (16.1)	124 (13.0)	404 (42.4)
Non-smoker	1645	143 (8.7)	120 (7.3)	121 (7.4)	125 (7.6)	339 (20.6)
*p*		<0.001	<0.001	<0.001	<0.001	<0.001

**Table 5:** Elemental carbon exposure and age of Western Australian miners by status of respiratory symptoms.

	Status of Respiratory Symptom		*p*
	No	Yes	
Cough			
*n*	2191	407	
Age, mean (SD)	33.8 (10.1)	36.4 (9.96)	<0.001
EC, median (IQR)	0.065 (0.074)	0.074 (0.079)	0.018
Phlegm			
*n*	2295	303	
Age, mean (SD)	33.9 (10.1)	36.9 (9.49)	<0.001
EC, median (IQR)	0.065 (0.074)	0.076 (0.074)	0.019
Wheeze			
*n*	2324	274	
Age, mean (SD)	33.9 (10.1)	36.8 (9.48)	<0.001
EC, median (IQR)	0.066 (0.073)	0.068 (0.073)	0.826
Breathlessness			
*n*	2349	249	
Age, mean (SD)	33.7 (9.88)	39.6 (10.4)	<0.001
EC, median (IQR)	0.067 (0.073)	0.066 (0.061)	0.902
Any			
*n*	1855	743	
Age, mean (SD)	33.3 (9.98)	36.5 (9.98)	<0.001
EC, median (IQR)	0.065 (0.075)	0.069 (0.07)	0.062
